# Supplementary material for: Physiological roles of an Acinetobacter-specific σ factor
Source: mBio. 2025 May 19;16(6):e00968-25. doi: 10.1128/mbio.00968-25 (PMC12153292; doi:10.1128/mbio.00968-25)
Supplement: Supplemental Figures — Figures S1 to S14. [file mbio.00968-25-s0001.pdf]

Figure S1

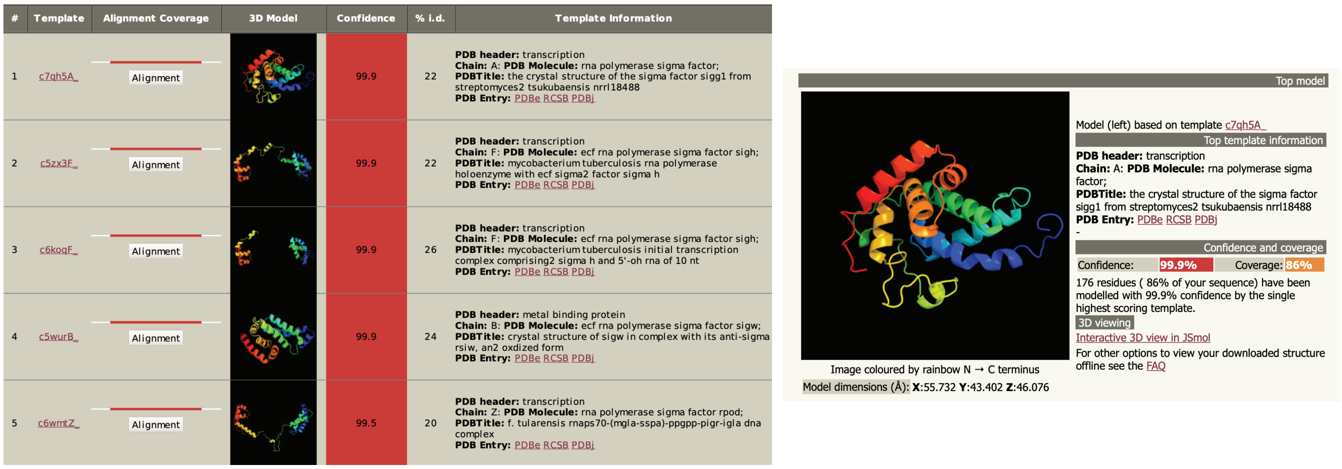

#	Template	Alignment Coverage	3D Model	Confidence	% i.d.	Template Information
1	c7qh5A_	Alignment		99.9	22	**PDB header:** transcription<br>**Chain:** A: **PDB Molecule:** rna polymerase sigma factor;<br>**PDBTitle:** the crystal structure of the sigma factor sigg1 from streptomyces2 tsukubaensis nrrl18488<br>**PDB Entry:** PDBe RCSB PDBj
2	c5zx3F_	Alignment		99.9	22	**PDB header:** transcription<br>**Chain:** F: **PDB Molecule:** ecf rna polymerase sigma factor sigh;<br>**PDBTitle:** mycobacterium tuberculosis rna polymerase holoenzyme with ecf sigma2 factor sigma h<br>**PDB Entry:** PDBe RCSB PDBj
3	c6koqF_	Alignment		99.9	26	**PDB header:** transcription<br>**Chain:** F: **PDB Molecule:** ecf rna polymerase sigma factor sigh;<br>**PDBTitle:** mycobacterium tuberculosis initial transcription complex comprising2 sigma h and 5'-oh rna of 10 nt<br>**PDB Entry:** PDBe RCSB PDBj
4	c5wurB_	Alignment		99.9	24	**PDB header:** metal binding protein<br>**Chain:** B: **PDB Molecule:** ecf rna polymerase sigma factor sigw;<br>**PDBTitle:** crystal structure of sigw in complex with its anti-sigma rsiw, an2 oxdized form<br>**PDB Entry:** PDBe RCSB PDBj
5	c6wmtZ_	Alignment		99.5	20	**PDB header:** transcription<br>**Chain:** Z: **PDB Molecule:** rna polymerase sigma factor rpod;<br>**PDBTitle:** f. tularensis rnaps70-(mgla-sspa)-ppgpp-pigr-igla dna complex<br>**PDB Entry:** PDBe RCSB PDBj

Top model

Image coloured by rainbow N → C terminus

Model dimensions (Å): **X**:55.732 **Y**:43.402 **Z**:46.076

Model (left) based on template c7qh5A_

Top template information

PDB header: transcription
Chain: A: **PDB Molecule:** rna polymerase sigma factor;
PDBTitle: the crystal structure of the sigma factor sigg1 from streptomyces2 tsukubaensis nrrl18488
PDB Entry: PDBe RCSB PDBj
-

Confidence and coverage

Confidence: **99.9%** Coverage: **86%**

176 residues (86% of your sequence) have been modelled with 99.9% confidence by the single highest scoring template.

3D viewing

Interactive 3D view in JSmol

For other options to view your downloaded structure offline see the FAQ

Figure S2

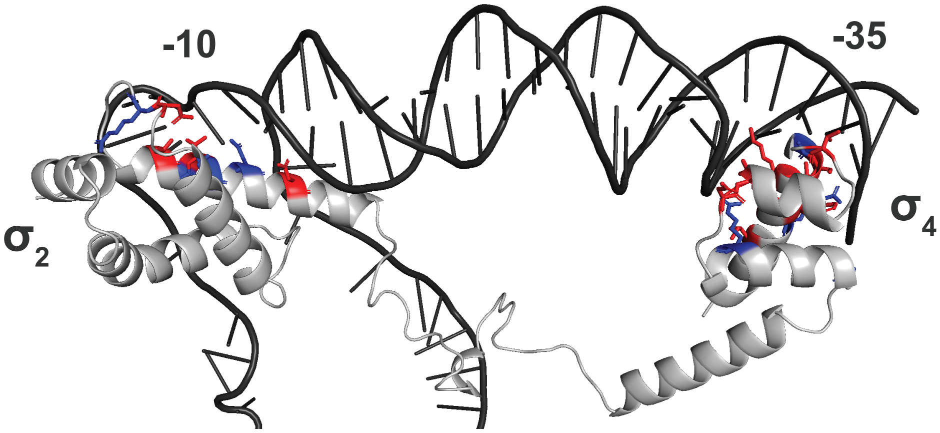

Blue = predicted DNA binding amino acids, conserved

Red = predicted DNA binding amino acids, not conserved

Figure S1. Mapping SigAb onto known protein structures confirms that SigAb is an ECF σ factor.

Template information for top 5 hits from Phyre2 result of SigAb structural modeling. 86% of the SigAb amino acid sequence is modeled with 99.9% confidence to the ECF sigma factors shown, but with low percent identity.

Figure S2. SigAb structural model traced onto RpoE holoenzyme structure.

SigAb structural model based on Phyre2 structural prediction and *E. coli* RpoE holoenzyme crystal structure (34). Predicted DNA binding residues are highlighted, with amino acids conserved in SigAb relative to RpoE in blue (R65, Y75, I77, N80, P139, R149, G168, R176) and amino acids not conserved in red (F64, W73, R76, N84, S155, Y156, P166, T169, R171, S172, R173, F175, R178).

Figure S3

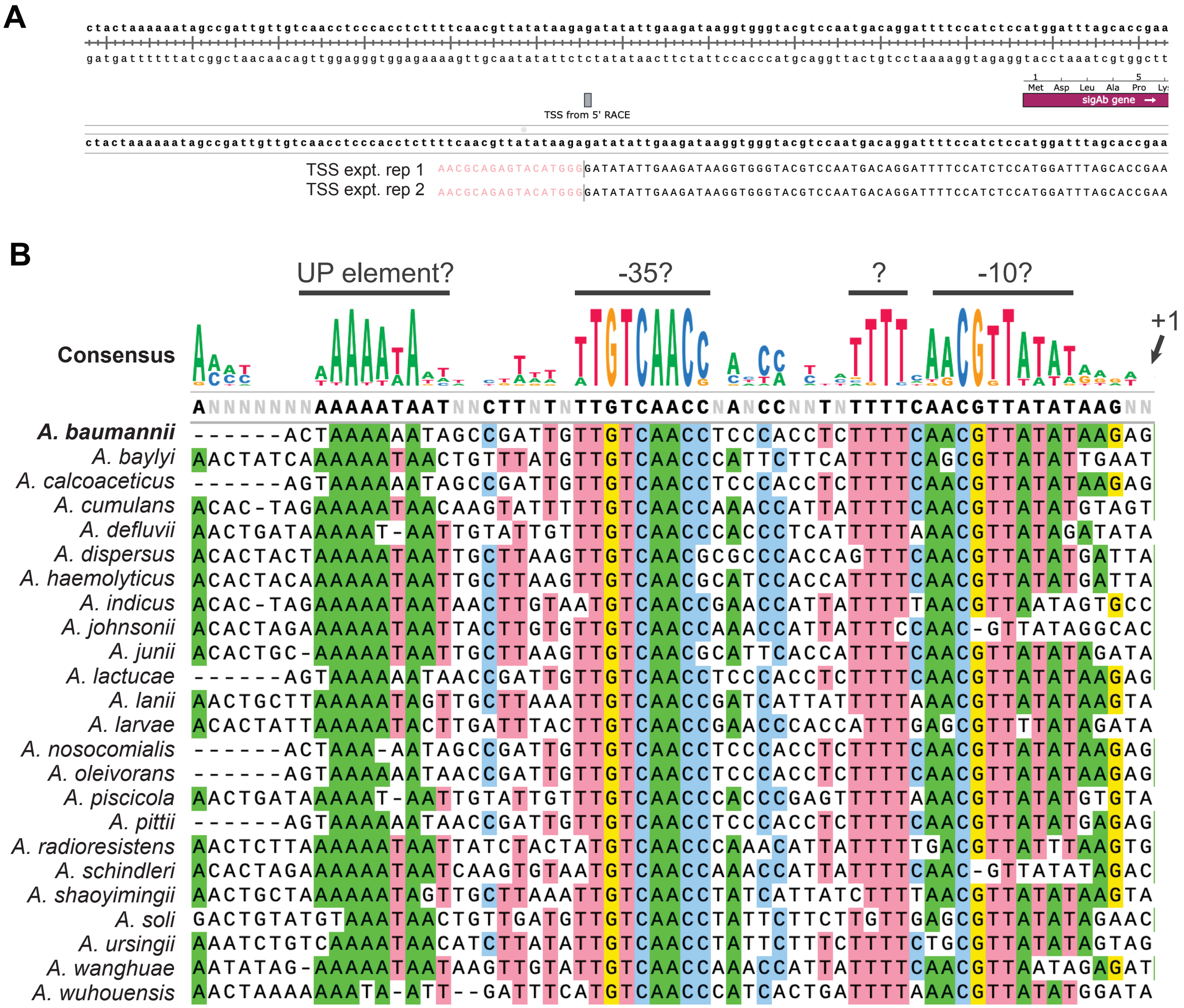
A
ctactaaaaaaatagccgattgttgtcaacctcccacctcttttcaacgttatataagagatatattgaagataaggtgggtacgtccaatgacaggattttccatctccatggatttagcaccgaa
Met Asp Leu Ala Pro Ly
sigAb gene
TSS from 5' RACE
TSS expt. rep 1
TSS expt. rep 2
GATATATTGAAGATAAGGTGGGTACGTCCAATGACAGGATTTTCCATCTCCATGGATTTAGCACCGAA
B
UP element?
-35?
?
-10?
+1
Consensus
ANNNNNNNAAAAATAATNNCTTNTNTTGTCAACCNANCCNNTNTTTTCAACGTTATATAAGNN
A. baumannii ------ACTAAAAAATAGCCGATTGTTGTCAACCTCCCACCTCTTTTCAACGTTATATAAGAG
A. baylyi AACTATCAAAAAATAACTGTTTATGTTGTCAACCCATTCTTCATTTTCAGCGTTATATTGAAT
A. calcoaceticus ------AGTAAAAAATAGCCGATTGTTGTCAACCTCCCACCTCTTTTCAACGTTATATAAGAG
A. cumulans ACAC-TAGAAAAATAACAAGTATTTTTGTCAACCAAACCATTATTTTCAACGTTATATGTAGT
A. defluvii AACTGATAAAAAT-AATTGTATTGTTTGTCAACCCACCCTCATTTTAAACGTTATAGATATA
A. dispersus ACACTACTAAAAATAATTGCTTAAGTTGTCAACGCGCCCACCAGTTTCAACGTTATATGATTA
A. haemolyticus ACACTACAAAAAATAATTGCTTAAGTTGTCAACGCATCCACCATTTTCAACGTTATATGATTA
A. indicus ACAC-TAGAAAAATAATAACTTGTAATGTCAACCGAACCATTATTTTTAACGTTAATAGTGCC
A. johnsonii ACACTAGAAAAAATAATTACTTGTGTTGTCAACCAAACCATTATTTCCAAC-GTTATAGGCAC
A. junii ACACTGC-AAAAATAATTGCTTAAGTTGTCAACGCATTCACCATTTTCAACGTTATATAGATA
A. lactucae ------AGTAAAAAATAACCGATTGTTGTCAACCTCCCACCTCTTTTCAACGTTATATAAGAG
A. lanii AACTGCTTAAAAATAGTTGCTTAAATTGTCAACCGATCATTATTTTTAAACGTTATATAAGTA
A. larvae ACACTATTAAAAATACTTGATTTACTTGTCAACCGAACCCACCATTTGAGCGTTTTATAGATA
A. nosocomialis ------ACTAAA-AATAGCCGATTGTTGTCAACCTCCCACCTCTTTTCAACGTTATATAAGAG
A. oleivorans ------AGTAAAAAATAACCGATTGTTGTCAACCTCCCACCTCTTTTCAACGTTATATAAGAG
A. piscicola AACTGATAAAAAT-AATTGTATTGTTTGTCAACCCACCCGAGTTTTAAACGTTATATGTGTA
A. pittii ------AGTAAAAAATAACCGATTGTTGTCAACCTCCCACCTCTTTTCAACGTTATATGAGAG
A. radioresistens AACTCTTAAAAAATAATTATCTACTATGTCAACCCAAACATTATTTTTGACGTTATTTAAGTG
A. schindleri ACACTAGAAAAAATAATCAAGTGTAATGTCAACCAAACCATTATTTTCAAC-GTTATATAGAC
A. shaoyimingii AACTGCTAAAAAATAGTTGCTTAAATTGTCAACCTATCATTATCTTTTAACGTTATATAAGTA
A. soli GACTGTATGTAAATAACTGTTGATGTTGTCAACCTATTCTTCTTGTTGAGCGTTATATAGAAC
A. ursingii AAATCTGTCAAAATAACATCTTATATTGTCAACCTATTCTTTCTTTTCTGCGTTATATAGTAG
A. wanghuae AATATAG-AAAAATAATAAGTTGTATTGTCAACCAAACCATTATTTTCAACGTTAATAGAGAT
A. wuhouensis AACTAAAAAAATA-ATT--GATTTCATGTCAACCCATCACTGATTTTAAACGTTATATGGATA

Figure S3. Prediction of key P_{sigAb} elements.

A 5’ end of *sigAb* transcript identified using 5’ RACE. RACE results from 2 replicates are shown.

B Alignment of DNA sequence upstream of putative *sigAb* transcription start site (TSS) across 24 representative *Acinetobacter* species. Predictions of potential promoter elements are labeled based on the regions highly conserved.

Figure S4

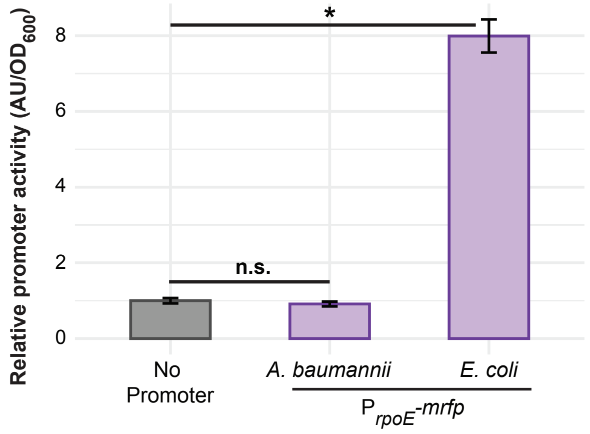

Figure S5

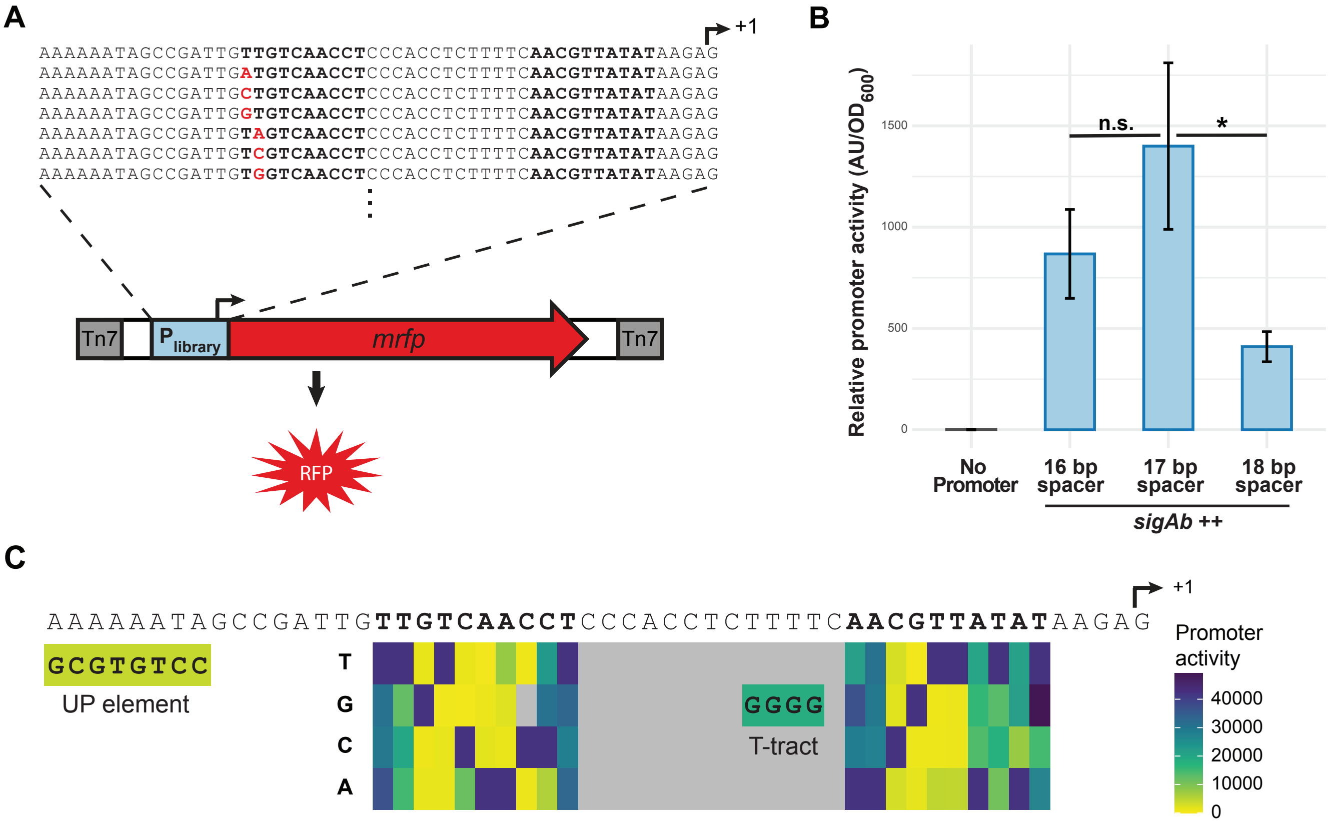

Figure S4. P$_{rpoE}$ does not function in WT *A. baumannii*.

Activity of *Eco*-RpoE promoter (P$_{rpoE}$) in *A. baumannii* or *E. coli*. Promoter activity is calculated as absorbance units (AU) normalized to OD_{600} and the no promoter control (n=3). Data are represented as the mean ± s.d. and significance was calculated with a two-tailed Student's *t*-test ($p<0.05$).

Figure S5. Promoter mutagenesis screen.

A Individual point mutations were introduced into P$_{sigAb}$ promoter and cloned as a library into Tn*7* fluorescent reporter. mRFP fluorescence was measured as promoter activity. **B** P$_{sigAb}$ activity with varying spacer lengths. Promoter activity is calculated as absorbance units (AU) normalized to OD_{600} and the no promoter control (n=3). Data are represented as the mean ± s.d. and significance was calculated with a two-tailed Student's *t*-test ($p<0.05$). **C** Compiled results for promoter activities from P$_{sigAb}$ mutagenesis screen. UP element and T-tract were both mutated as one chunk. Heatmap shown is median of n=5 assays for 1-13 biological replicates per mutation.

Figure S6

A

```
1:        -tattgctgacccaatggcgtcaacgagaaacctatattca-acgtttaagccaaa 54
2:        -ttcggcttttgcttcggggtcaacccagtttttgtcatg-tcgtttggtttcat 54
3:        -ttagtcccagataagtttgtcaacggcgttatcctcataa-tcgttgatcaagga 54
sigAb:    -aaaaaatagccgattgttgtcaacctcccacctcttttca-acgttatataagag 54
5:        -attgacccggcaaccttagtcaacgataaagtaaatagtt-acgttgaaaaggct 54
6:        -attgcaggtattggtcaagtcaacggcatcacttgcatga-tcgttgctaacgat 54
7:        -ttcgtaaagttgctccaggtcaaccgatgacaatggatta-tcgttcaaatcgag 54
sabS:     -atttttttcatttttctgtcaaccaatcctgatctcttg-acgttatatagggt 54
9:        -taaagttgttttaccatggtcaacgtgaccgattgtaccc-acgtttacgtgtgg 54
10:       -caatgcgatagcagcaacgtcaacgttaccggtacgttca-ccgttaccaaatac 54
11:       -taaagttgttttaccatggtcaacgtgaccgattgtaccc-acgtttacgtgtgg 54
12:       --cttcaggtttagaagcggtcaacatggcagccatgaaagttcgttctggttggg 54
13:       --tctagaacttgcattaagtcaacaaggtgagatcgcaatttcgttctctggagc 54
14:       --ttttaagatttgggttagtcaacaaaccataacgatagaaacgttcaatatcgt 54
15:       ttccaaggtggtgatgctggtcaacaaaaagcagacgatgg--cgttgtagatgct 54
16:       tggcaatcttggttgcagcgtcaactcgtgtctagttctcg--cgttgagcaaaag 54
relA:     aaaactaaacaatgctgatgtcaaccaattaagacaatttg--cgttacacaaagg 54
                             ******                  ****
```

B

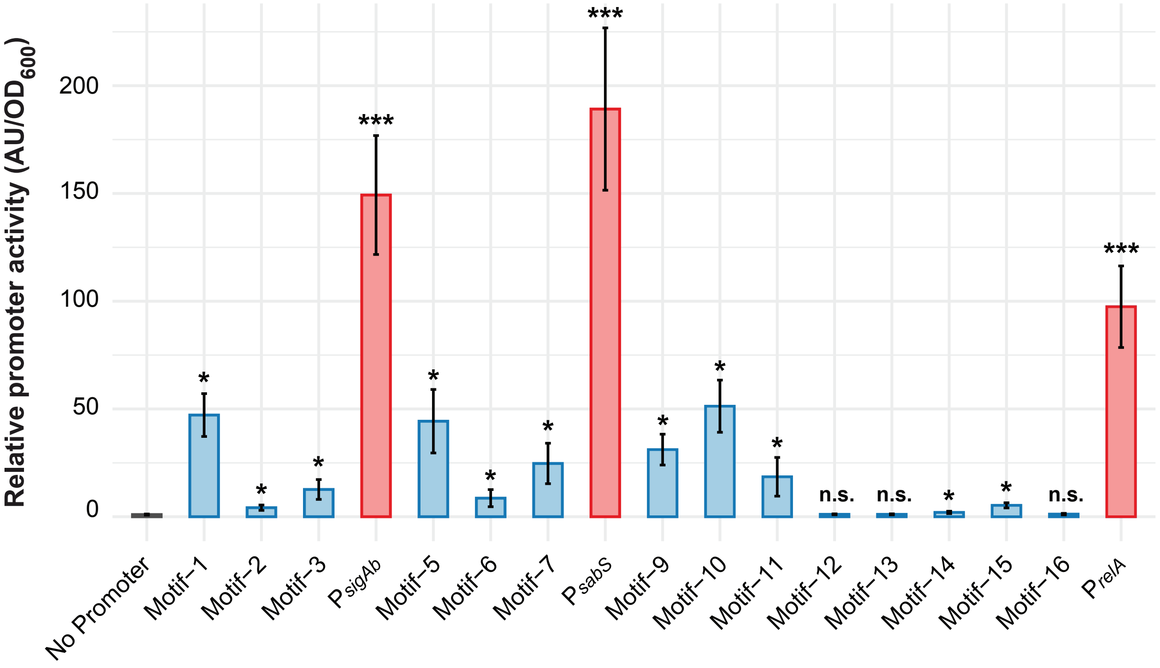

Figure S6. 17 putative SigAb-dependent promoter motifs in *A. baumannii*.

A Alignment of all 17 putative P_{sigAb} motifs found in *A. baumannii*. 3 of the motifs (P_{sigAb}, P_{relA}, and P_{16}) are upstream of protein coding genes; the other motifs are either inter or intragenic. **B** Promoter activity of putative SigAb-dependent motifs from (a) in mRFP reporter in strains containing *sigAb* overexpression vector induced with 50 µM IPTG. Promoter activity is calculated as absorbance units (AU) normalized to OD_{600} and the no promoter control (n=3). Data are represented as the mean ± s.d. and significance was calculated with a two-tailed Student's *t*-test (p<0.05). Significance relative to No Promoter control is indicated by one star (*). Activity of P_{sigAb}, P_{sabS}, and P_{relA} (red bars) are significantly different than each of the other motifs (***).

Figure S7

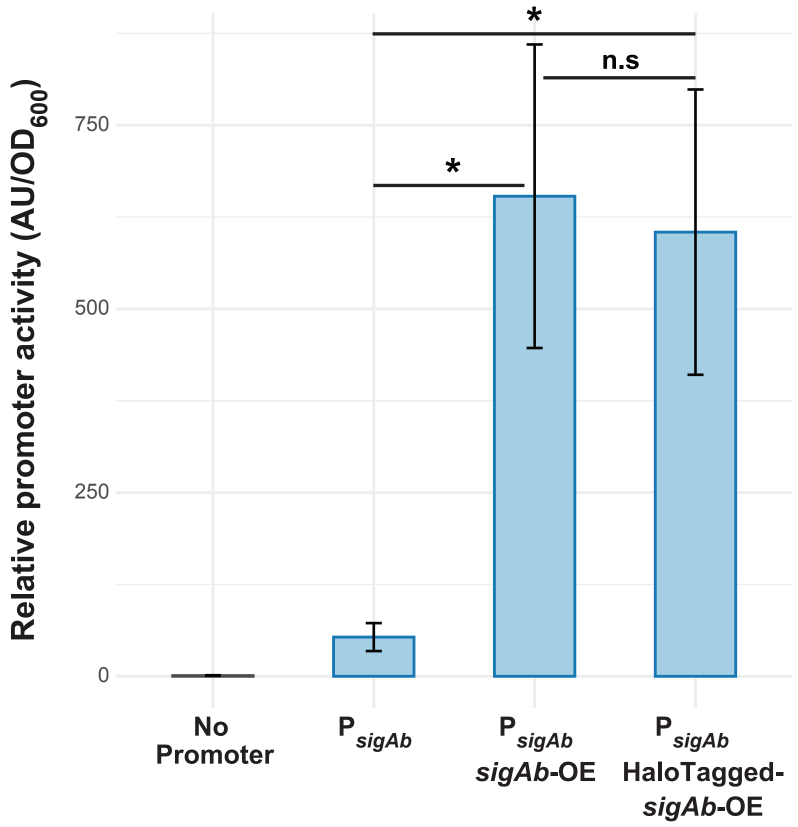

Relative promoter activity (AU/OD600)
750
500
250
0
*
n.s
*
No Promoter
PsigAb
PsigAb sigAb-OE
PsigAb HaloTagged-sigAb-OE

Figure S7. Tagged SigAb retains its function.

P_{sigAb} activity with either WT-SigAb overexpression (OE) or tagged-SigAb OE from IPTG-inducible replicative expression vectors induced with 50 μM IPTG. *sigAb* was N-terminally tagged with HaloTag. Promoter activity is calculated as absorbance units (AU) normalized to OD_{600} and the no promoter control (n=3). Data are represented as the mean ± s.d. and significance was calculated with a two-tailed Student's *t*-test ($p<0.05$).

Figure S8

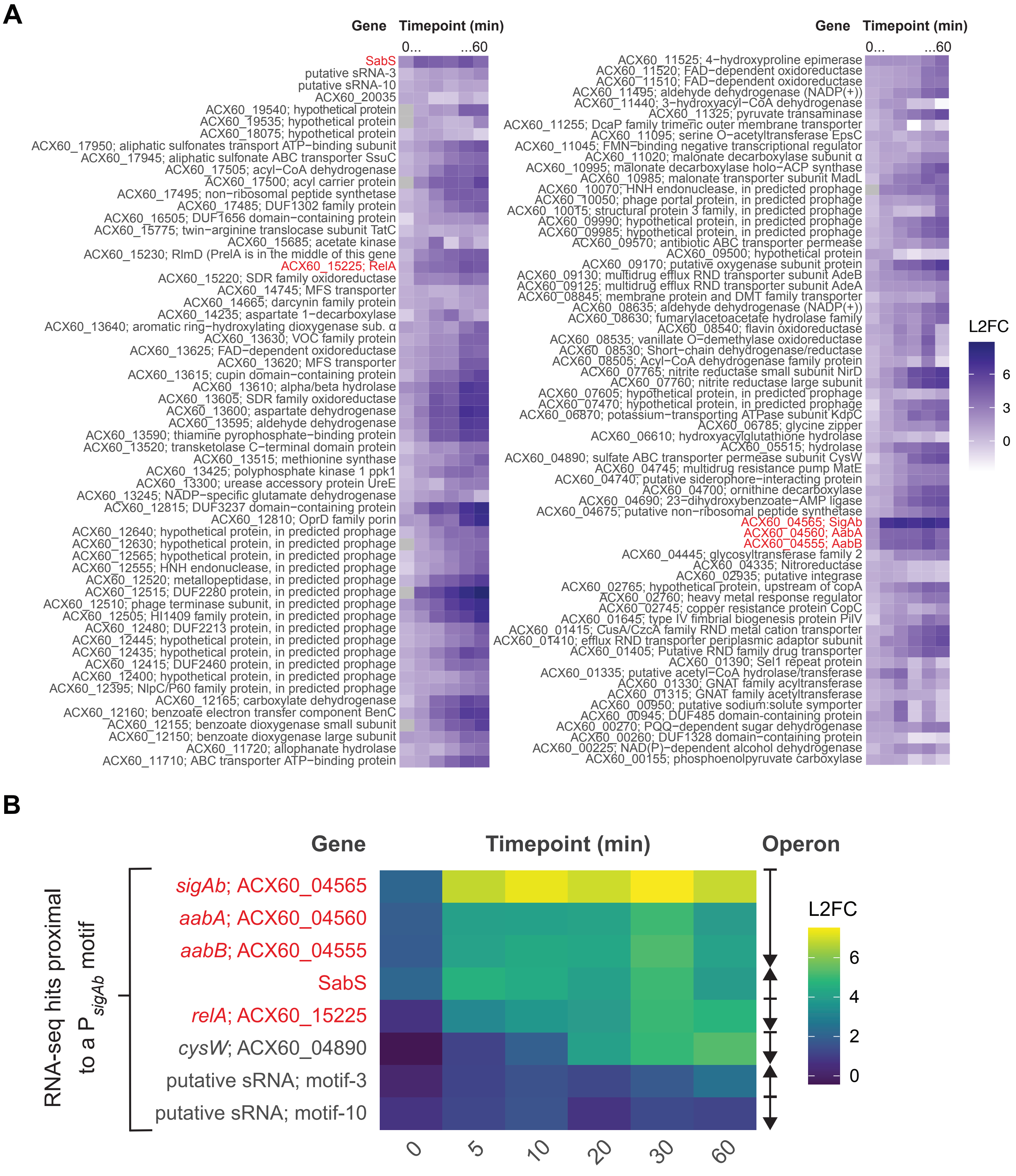

A
Gene
Timepoint (min)
0...
...60
SabS
putative sRNA-3
putative sRNA-10
ACX60_20035
ACX60_19540; hypothetical protein
ACX60_19535; hypothetical protein
ACX60_18075; hypothetical protein
ACX60_17950; aliphatic sulfonates transport ATP−binding subunit
ACX60_17945; aliphatic sulfonate ABC transporter SsuC
ACX60_17505; acyl−CoA dehydrogenase
ACX60_17500; acyl carrier protein
ACX60_17495; non−ribosomal peptide synthetase
ACX60_17485; DUF1302 family protein
ACX60_16505; DUF1656 domain−containing protein
ACX60_15775; twin−arginine translocase subunit TatC
ACX60_15685; acetate kinase
ACX60_15230; RlmD (PrelA is in the middle of this gene
ACX60_15225; RelA
ACX60_15220; SDR family oxidoreductase
ACX60_14745; MFS transporter
ACX60_14665; darcynin family protein
ACX60_14235; aspartate 1−decarboxylase
ACX60_13640; aromatic ring−hydroxylating dioxygenase sub. α
ACX60_13630; VOC family protein
ACX60_13625; FAD−dependent oxidoreductase
ACX60_13620; MFS transporter
ACX60_13615; cupin domain−containing protein
ACX60_13610; alpha/beta hydrolase
ACX60_13605; SDR family oxidoreductase
ACX60_13600; aspartate dehydrogenase
ACX60_13595; aldehyde dehydrogenase
ACX60_13590; thiamine pyrophosphate−binding protein
ACX60_13520; transketolase C−terminal domain protein
ACX60_13515; methionine synthase
ACX60_13425; polyphosphate kinase 1 ppk1
ACX60_13300; urease accessory protein UreE
ACX60_13245; NADP−specific glutamate dehydrogenase
ACX60_12815; DUF3237 domain−containing protein
ACX60_12810; OprD family porin
ACX60_12640; hypothetical protein, in predicted prophage
ACX60_12630; hypothetical protein, in predicted prophage
ACX60_12565; hypothetical protein, in predicted prophage
ACX60_12555; HNH endonuclease, in predicted prophage
ACX60_12520; metallopeptidase, in predicted prophage
ACX60_12515; DUF2280 protein, in predicted prophage
ACX60_12510; phage terminase subunit, in predicted prophage
ACX60_12505; HI1409 family protein, in predicted prophage
ACX60_12480; DUF2213 protein, in predicted prophage
ACX60_12445; hypothetical protein, in predicted prophage
ACX60_12435; hypothetical protein, in predicted prophage
ACX60_12415; DUF2460 protein, in predicted prophage
ACX60_12400; hypothetical protein, in predicted prophage
ACX60_12395; NlpC/P60 family protein, in predicted prophage
ACX60_12165; carboxylate dehydrogenase
ACX60_12160; benzoate electron transfer component BenC
ACX60_12155; benzoate dioxygenase small subunit
ACX60_12150; benzoate dioxygenase large subunit
ACX60_11720; allophanate hydrolase
ACX60_11710; ABC transporter ATP−binding protein
Gene
Timepoint (min)
0...
...60
ACX60_11525; 4−hydroxyproline epimerase
ACX60_11520; FAD−dependent oxidoreductase
ACX60_11510; FAD−dependent oxidoreductase
ACX60_11495; aldehyde dehydrogenase (NADP(+))
ACX60_11440; 3−hydroxyacyl−CoA dehydrogenase
ACX60_11325; pyruvate transaminase
ACX60_11255; DcaP family trimeric outer membrane transporter
ACX60_11095; serine O−acetyltransferase EpsC
ACX60_11045; FMN−binding negative transcriptional regulator
ACX60_11020; malonate decarboxylase subunit α
ACX60_10995; malonate decarboxylase holo−ACP synthase
ACX60_10985; malonate transporter subunit MadL
ACX60_10070; HNH endonuclease, in predicted prophage
ACX60_10050; phage portal protein, in predicted prophage
ACX60_10015; structural protein 3 family, in predicted prophage
ACX60_09990; hypothetical protein, in predicted prophage
ACX60_09985; hypothetical protein, in predicted prophage
ACX60_09570; antibiotic ABC transporter permease
ACX60_09500; hypothetical protein
ACX60_09170; putative oxygenase subunit protein
ACX60_09130; multidrug efflux RND transporter subunit AdeB
ACX60_09125; multidrug efflux RND transporter subunit AdeA
ACX60_08845; membrane protein and DMT family transporter
ACX60_08635; aldehyde dehydrogenase (NADP(+))
ACX60_08630; fumarylacetoacetate hydrolase family
ACX60_08540; flavin oxidoreductase
ACX60_08535; vanillate O−demethylase oxidoreductase
ACX60_08530; Short−chain dehydrogenase/reductase
ACX60_08505; Acyl−CoA dehydrogenase family protein
ACX60_07765; nitrite reductase small subunit NirD
ACX60_07760; nitrite reductase large subunit
ACX60_07605; hypothetical protein, in predicted prophage
ACX60_07470; hypothetical protein, in predicted prophage
ACX60_06870; potassium−transporting ATPase subunit KdpC
ACX60_06785; glycine zipper
ACX60_06610; hydroxyacylglutathione hydrolase
ACX60_05515; hydrolase
ACX60_04890; sulfate ABC transporter permease subunit CysW
ACX60_04745; multidrug resistance pump MatE
ACX60_04740; putative siderophore−interacting protein
ACX60_04700; ornithine decarboxylase
ACX60_04690; 23−dihydroxybenzoate−AMP ligase
ACX60_04675; putative non−ribosomal peptide synthetase
ACX60_04565; SigAb
ACX60_04560; AabA
ACX60_04555; AabB
ACX60_04445; glycosyltransferase family 2
ACX60_04335; Nitroreductase
ACX60_02935; putative integrase
ACX60_02765; hypothetical protein, upstream of copA
ACX60_02760; heavy metal response regulator
ACX60_02745; copper resistance protein CopC
ACX60_01645; type IV fimbrial biogenesis protein PilV
ACX60_01415; CusA/CzcA family RND metal cation transporter
ACX60_01410; efflux RND transporter periplasmic adaptor subunit
ACX60_01405; Putative RND family drug transporter
ACX60_01390; Sel1 repeat protein
ACX60_01335; putative acetyl−CoA hydrolase/transferase
ACX60_01330; GNAT family acyltransferase
ACX60_01315; GNAT family acetyltransferase
ACX60_00950; putative sodium:solute symporter
ACX60_00945; DUF485 domain−containing protein
ACX60_00270; PQQ−dependent sugar dehydrogenase
ACX60_00260; DUF1328 domain−containing protein
ACX60_00225; NAD(P)−dependent alcohol dehydrogenase
ACX60_00155; phosphoenolpyruvate carboxylase
L2FC
6
3
0
B
Gene
Timepoint (min)
Operon
RNA-seq hits proximal to a P sigAb motif
sigAb; ACX60_04565
aabA; ACX60_04560
aabB; ACX60_04555
SabS
relA; ACX60_15225
cysW; ACX60_04890
putative sRNA; motif-3
putative sRNA; motif-10
0
5
10
20
30
60
L2FC
6
4
2
0

Figure S8. Genes upregulated by *sigAb* overexpression.

A Heatmap of all significant hits ($Log_2FC > 1$ and $FDR < 0.05$) at 5 min timepoint for *sigAb* overexpression RNA-seq data compared to empty vector control. **B** Heatmap of RNA-seq hits with putative SigAb promoter motifs ($Log_2FC > 1$ and $FDR < 0.05$ at 5 min timepoint). Genes in red are also ChIP-seq hits and have promoter motifs highly conserved across *Acinetobacter* sp.

Figure S9

A

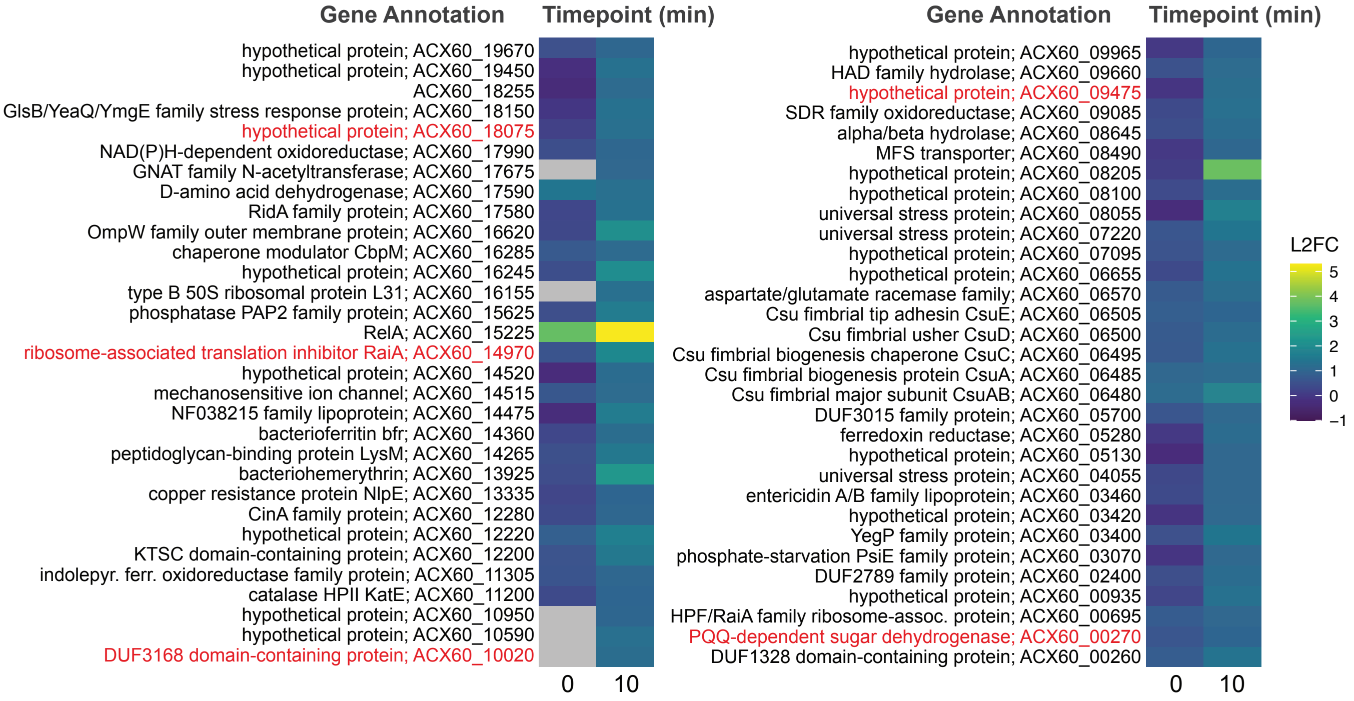

B

Significant upregulated hits after 10 min of *relA* overexpression

Group	STRING cluster	# genes
Nitrogen compound metabolic process	CL:98	9
Potassium ion transport and electron transport chain	CL:5548	9
Outer membrane and integral component of membrane	CL:3475	9
Metabolism and oxidoreductase activity	CL:1579	5
Membrane and regulation of cellular process	CL:3469	3
Hydrolase activity	CL:5413	3
Amino acid permease	CL:5880	2
Sulfur relay system and oxidoreductase activity	CL:3135	2
Other	---	9
No annotations	---	12
Total		62

Figure S9. Genes upregulated by *relA* overexpression.

A Heatmap of genes significantly upregulated ($Log_2FC > 1$, $FDR < 0.05$, T=10 min) in *relA* overexpression RNA-seq experiment compared to empty vector control. Genes labeled in red are also top hits (T=10 min) in *sigAb* RNA-seq experiment. **B** Table of gene set enrichments for genes significantly upregulated after 10 min of induction.

Figure S10

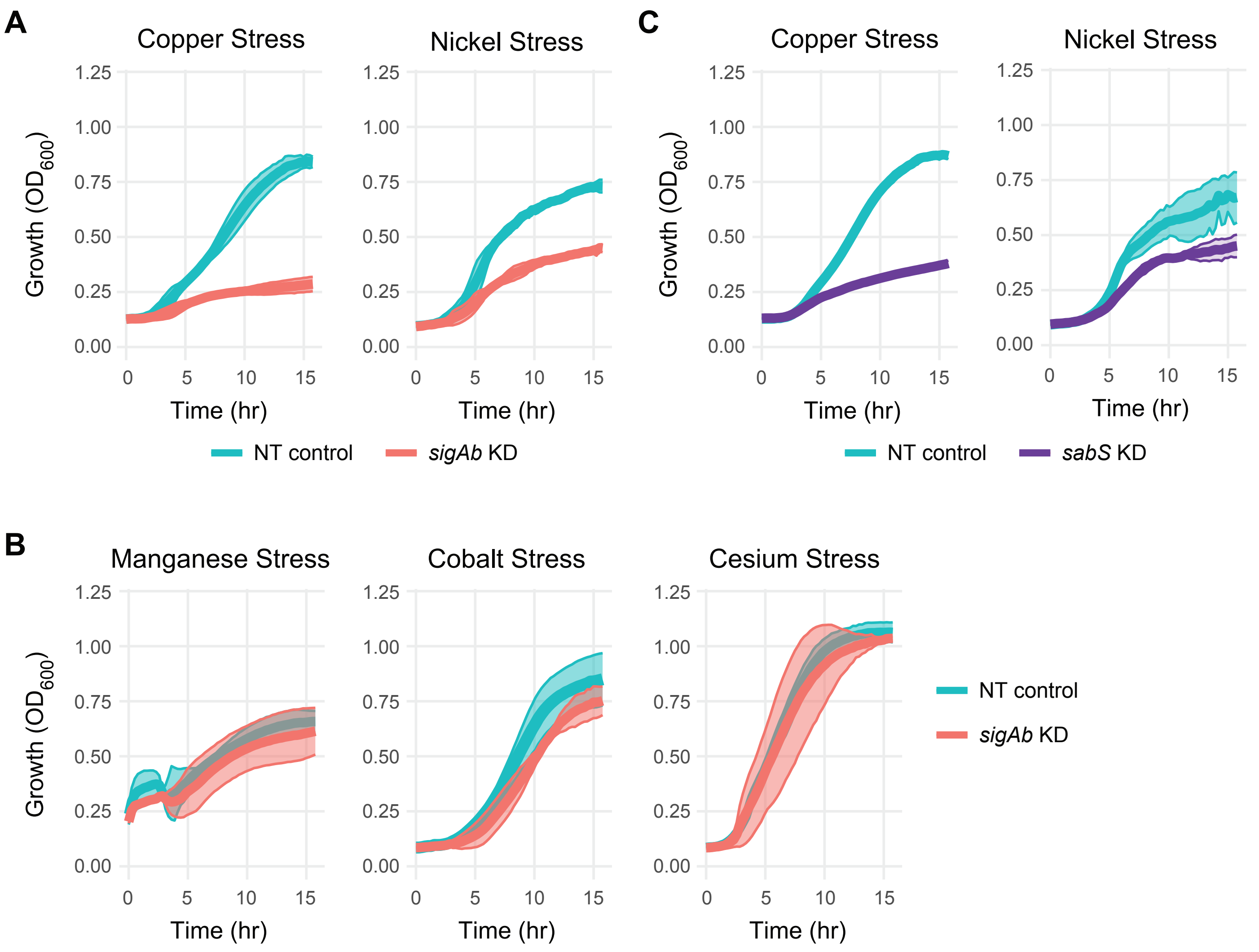
A
Copper Stress
Nickel Stress
C
Copper Stress
Nickel Stress
Growth (OD_{600})
Time (hr)
NT control
sigAb KD
NT control
sabS KD
B
Manganese Stress
Cobalt Stress
Cesium Stress
NT control
sigAb KD

Figure S10. *sigAb* knockdown strain is sensitive to specific heavy metal stresses.

A Conditions with SigAb-dependent phenotypes. Growth curves plotted as OD_{600} over Time (hr) for Copper and Nickel stresses. Nontargeting (NT) control strain is in blue and *sigAb* knockdown (KD) strain in red. Data are represented as mean ± s.d. (n=2). **B** Conditions with SabS-dependent phenotypes. Growth curves plotted as OD_{600} over Time (hr) for Copper and Nickel stresses. NT control strain is in blue and *sabS* KD strain in purple. Data are represented as mean ± s.d. (n=2). **C** Growth curves for representative conditions that don't affect *sigAb* KD growth relative to NT control using Biolog Phenotype Microarray plates PM13 and PM16. Data are represented as mean ± s.d. (n=2).

Figure S11

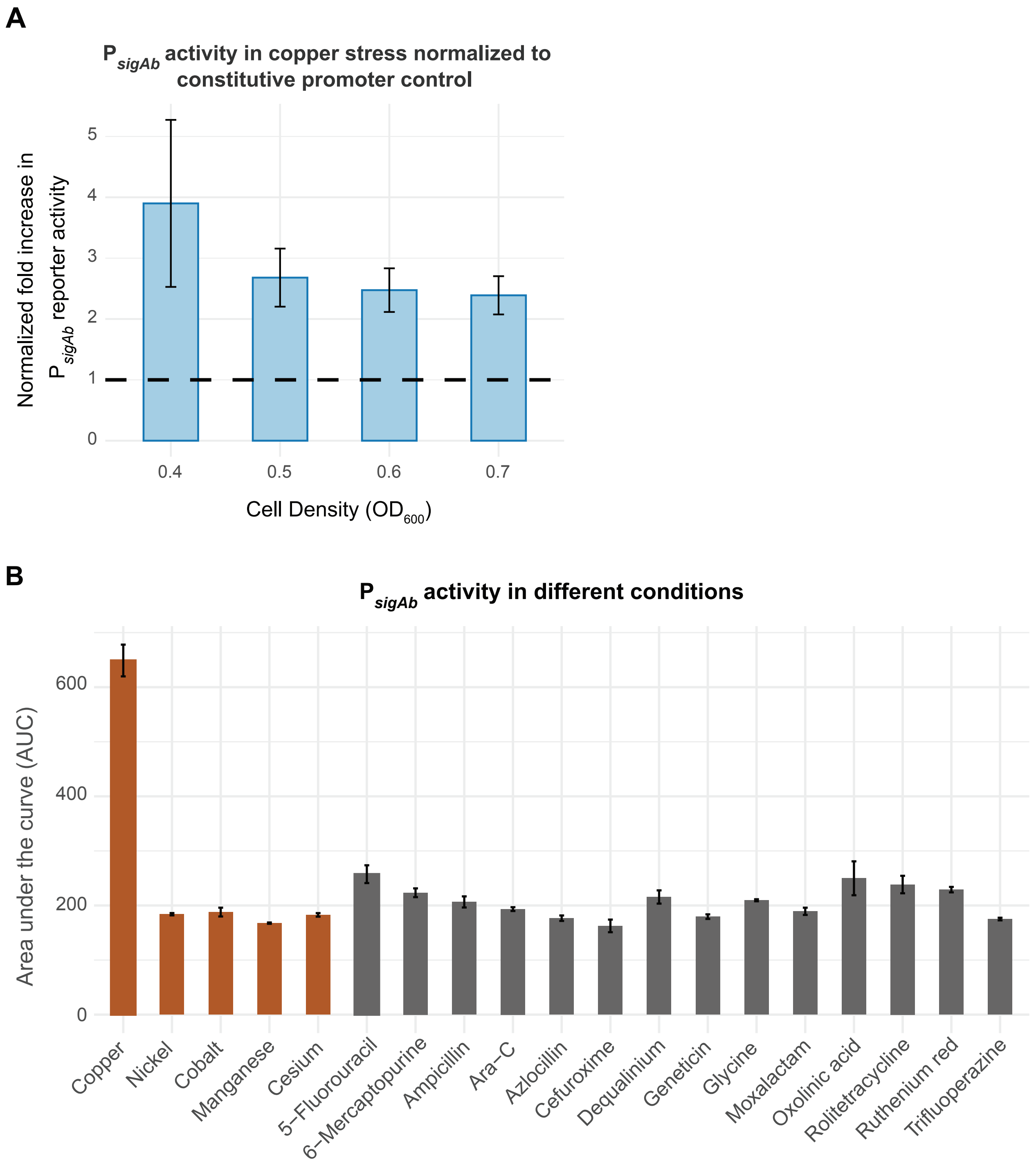
A
P_{sigAb} activity in copper stress normalized to constitutive promoter control
Normalized fold increase in P_{sigAb} reporter activity
0
1
2
3
4
5
0.4
0.5
0.6
0.7
Cell Density (OD_{600})
B
P_{sigAb} activity in different conditions
Area under the curve (AUC)
0
200
400
600
Copper
Nickel
Cobalt
Manganese
Cesium
5–Fluorouracil
6–Mercaptopurine
Ampicillin
Ara–C
Azlocillin
Cefuroxime
Dequalinium
Geneticin
Glycine
Moxalactam
Oxolinic acid
Rolitetracycline
Ruthenium red
Trifluoperazine

Figure S11. Copper is an inducing signal of SigAb.

A P_{sigAb}-*mrfp* reporter activity normalized to P_{lacUV5}-*mrfp* constitutive reporter control in rich defined medium with copper ($CuSO_4$) stress (n=5). Data are represented as the fold change (FC) ± s.d. and a horizontal dotted line at FC=1 indicates normalized reporter activity without copper stress. **B** Area under the curve (AUC) values quantifying induction curves (from Fig. 5d). Metal stresses are colored in brown and antibiotic stresses in grey. Data are represented as mean ± s.d. (n=2).

Figure S12

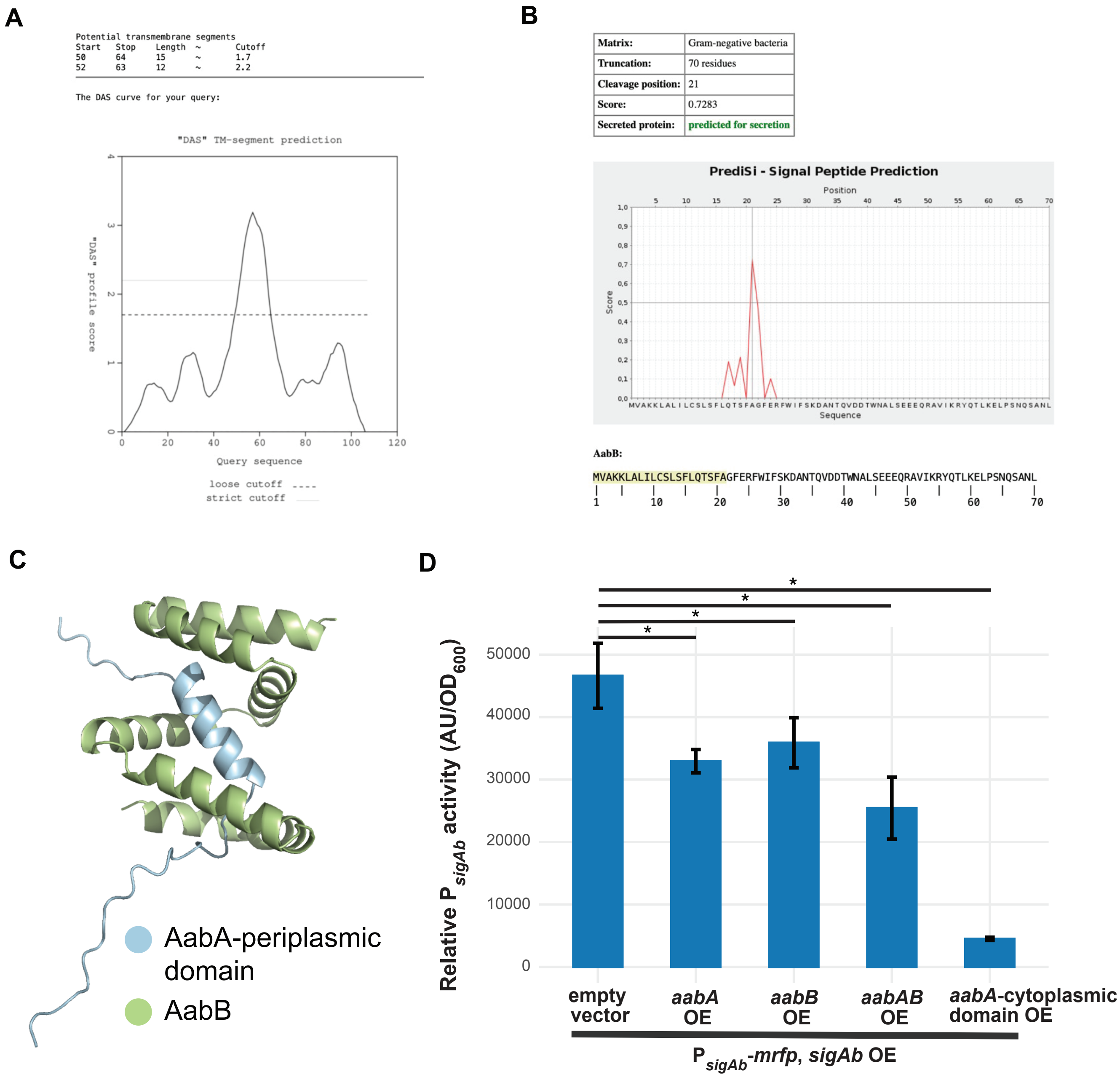
A
Potential transmembrane segments
Start Stop Length ~ Cutoff
50 64 15 ~ 1.7
52 63 12 ~ 2.2
The DAS curve for your query:
"DAS" TM-segment prediction
"DAS" profile score
Query sequence
loose cutoff
strict cutoff
B
Matrix: Gram-negative bacteria
Truncation: 70 residues
Cleavage position: 21
Score: 0.7283
Secreted protein: predicted for secretion
PrediSi - Signal Peptide Prediction
Position
Score
Sequence
AabB:
MVAKKLALILCSLSFLQTSFAGFERFWIFSKDANTQVDDTWNALSEEEQRAVIKRYQTLKELPSNQSANL
C
AabA-periplasmic domain
AabB
D
Relative P sigAb activity (AU/OD600)
empty vector
aabA OE
aabB OE
aabAB OE
aabA-cytoplasmic domain OE
P sigAb-mrfp, sigAb OE

Figure S12. SigAb-AabA-AabB protein features.

A Transmembrane domain of AabA predicted using TMHMM 2.0. **B** Signal recognition sequence of AabB predicted using SignalP 6.0. **C** AabA-AabB structural interaction model. Model predicted using AlphaFold2 run on COSMIC2 cloud platform. **D** mRFP reporter assay for P_{sigAb} activity in *E. coli* strains harboring overexpression vectors with *aabA*, *aabB*, both *aabA* and *aabB*, just the *aabA* cytoplasmic domain, or an empty vector control. All strains contain a *sigAb* overexpression vector. Promoter activity is calculated as absorbance units (AU) normalized to OD_{600} (n=6). Data are represented as the mean ± s.d. and significance was calculated with a two-tailed Student's *t*-test ($p<0.05$).

Figure S13

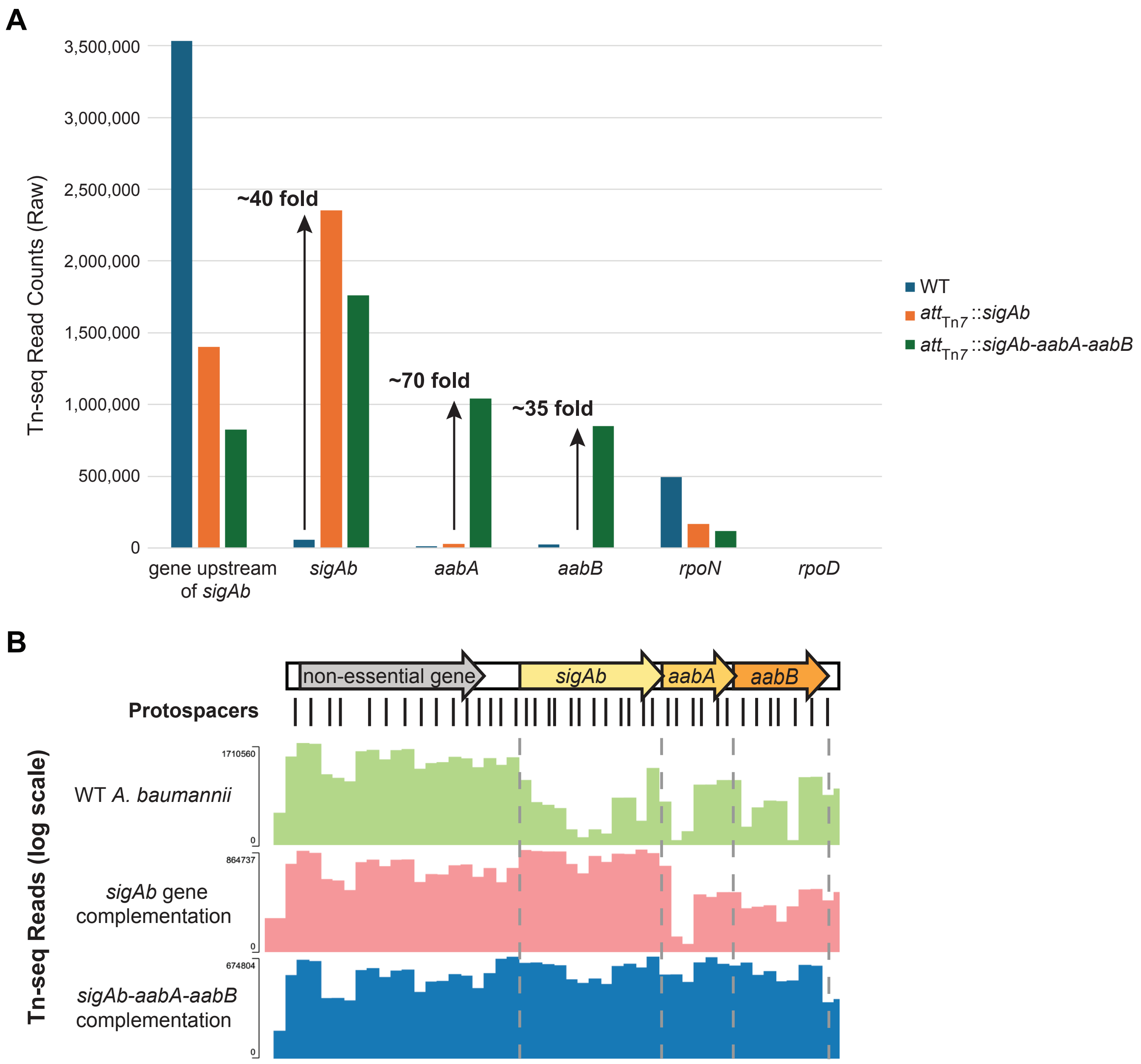

A
3,500,000
3,000,000
2,500,000
2,000,000
1,500,000
1,000,000
500,000
0
Tn-seq Read Counts (Raw)
~40 fold
~70 fold
~35 fold
gene upstream of sigAb
sigAb
aabA
aabB
rpoN
rpoD
WT
att Tn7 ::sigAb
att Tn7 ::sigAb-aabA-aabB
B
non-essential gene
sigAb
aabA
aabB
Protospacers
Tn-seq Reads (log scale)
WT A. baumannii
sigAb gene complementation
sigAb-aabA-aabB complementation

Figure S13. Quantification of CRISPRt total insertion reads in each gene.

A Sum of total CRISPRt read counts per gene in 3 different strain backgrounds: WT (blue), *sigAb* gene complementation (orange), or *sigAb* operon complementation (green). Protospacers targeting each gene are pooled together as 1 library. Decrease in read counts in the non-essential genes "gene upstream of *sigAb*" and *rpoN* are due to the increase in *sigAb*, *aabA*, and *aabB* read counts (complementation strains, orange and green bars). **B** CRISPRt insertions within the *sigAb* operon in either WT *A. baumannii* (green), a strain harboring *sigAb* gene duplication in att_{Tn7} site (pink), or a strain harboring *sigAb* operon duplication in att_{Tn7} site (blue). Normalized Tn-seq reads are shown on a log scale.

Figure S14

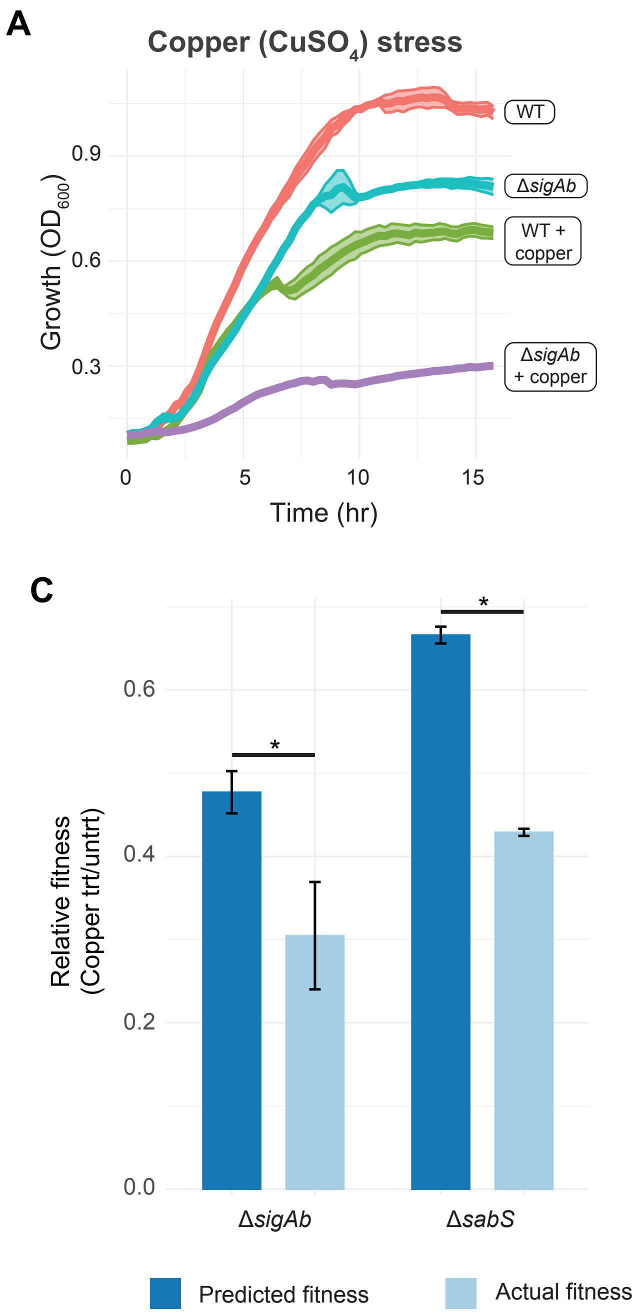
A
Copper ($CuSO_4$) stress
Growth (OD_{600})
0.3
0.6
0.9
0
5
10
15
Time (hr)
WT
ΔsigAb
WT + copper
ΔsigAb + copper
B
Copper ($CuSO_4$) stress
Growth (OD_{600})
0.3
0.6
0.9
0
5
10
15
Time (hr)
WT
ΔsabS
WT + copper
ΔsabS + copper
C
Relative fitness (Copper trt/untrt)
0.0
0.2
0.4
0.6
*
*
ΔsigAb
ΔsabS
Predicted fitness
Actual fitness

Figure S14. Δ*sigAb* and Δ*sabS* strains are sensitive to copper stress.

A Growth curves plotted as OD_{600} over time (hr) of *sigAb* deletion (Δ*sigAb*) strain and wild-type (WT) control in rich defined medium with 250 μg/mL $CuSO_4$ stress (n=3). Data are represented as mean ± s.d. for WT with no stress (red), Δ*sigAb* with no stress (blue), WT with copper stress (green), and Δ*sigAb* with copper stress (purple). **B** Growth curves plotted as OD_{600} over time (hr) of *sabS* deletion (Δ*sabS*) strain and WT control in rich defined medium with 250 μg/mL $CuSO_4$ stress (n=3). Data are represented as mean ± s.d. for WT with no stress (red), Δ*sabS* with no stress (blue), WT with copper stress (green), and Δ*sabS* with copper stress (purple). **C** Fitness of Δ*sigAb* and Δ*sabS* strains in rich defined medium with copper stress normalized to an unstressed control. Data are represented as mean relative fitness ± s.d. for predicted (dark blue) and experimental (light blue) fitness. Predicted fitness is calculated using the multiplicative model (fitness of WT in copper × fitness of deletion strain).
